# Associations of Age, Preinjury Morbidity, Injury Severity, and Cognitive Impairment With Mortality and Length of Stay in Trauma Consultation Patients: A Retrospective Study

**DOI:** 10.7759/cureus.69661

**Published:** 2024-09-18

**Authors:** C. Michael Dunham, Gregory S Huang, Elisha A Chance, Barbara M Hileman

**Affiliations:** 1 Trauma, Critical Care, and General Surgery, Mercy Health - St. Elizabeth Youngstown Hospital, Youngstown, USA; 2 Trauma and Neuroscience Research, Mercy Health - St. Elizabeth Youngstown Hospital, Youngstown, USA

**Keywords:** cigarette smoking, consult patients, glasgow coma scale, injury severity score, intracranial hemorrhage, preexisting medical conditions, trauma centers, trauma mortality

## Abstract

Background

To the best of our knowledge, we have found no trauma consultation study investigating Injury Severity Score (ISS) ≥16, Glasgow Coma Scale score (GCS), intracranial hemorrhage (ICH), age, preexisting medical conditions (PEMC), and smoking as risk conditions for mortality.

Objective

We aimed to assess ISS ≥16 and other postinjury and preinjury conditions for associations with death and adverse outcomes (AO).

Methodology

Consecutive consultations of patients admitted to a trauma center over 18 months were investigated. Data were obtained from the trauma registry and the electronic medical record. AO were death, intensive care unit stay of two days or more, or hospital stay exceeding five days.

Results

Among 1,031 trauma consultations, 28 patients (2.7%) died and 258 (25.0%) had AO. The proportion of ISS ≥16 was greater with death (53.6% (15/28)) than with survival (20.2% (203/1,003); p<0.0001). Of 218 patients with ISS ≥16, 93.1% (n = 203) survived, whereas 46.4% (13/28) died with an ISS <16. The area under the receiver operating characteristic curve for ISS ≥16 and the death relationship was 0.7 (p<0.001). The proportion of GCS <15 was greater with death (42.9% (12/28)) than with survival (13.1% (131/1,003); p<0.0001). The incidence of ICH was greater with death (57.1% (16/28)) than with survival (32.5% (326/1,003); p=0.0063). The incidence of age ≥70 was greater with death (89.3% (25/28)) than with survival (48.2% (483/1,003); p<0.0001). The proportion of PEMC was greater with death (85.7% (24/28)) than with survival (50.8% (509/1,003); p=0.0002). The proportion of smoking history was similar with death (50.0% (14/28)) and survival (52.5% (527/1,003); p=0.7905). Death had independent associations with age (p=0.0019), GCS (p<0.0001), ISS ≥16 (p=0.0074), and PEMC (p=0.0137). AO had univariate associations with ISS ≥16 (p<0.0001), GCS <15 (p<0.0001), ICH (p=0.0004), and PEMC (p=0.0002). Area under the receiver operating characteristic curve for ISS ≥16 and the AO relationship was 0.6 (p<0.001). AO had independent associations with GCS (p<0.0001), ISS ≥16 (p<0.0001), and PEMC (p=0.0005).

Conclusions

ISS ≥16 alone is marginally accurate for classifying trauma consultation patients who died or had AO. Other postinjury and preinjury conditions, such as GCS, ICH, age, and PEMC, should also be considered when assessing one’s risk of death and AO.

## Introduction

Numerous trauma investigations have shown that hospital mortality or unfavorable outcomes post-discharge have been associated with an increased Injury Severity Score (ISS), decreased Glasgow Coma Scale score (GCS), intracranial hemorrhage (ICH), increased age, and preexisting medical conditions (PEMCs). The severity of anatomic injury, using the ISS, has been shown to be associated with in-hospital mortality [1-4]. One of these investigations formulated a meta-analysis [3] and one study was based on a large database [1]. The other studies provided median ISS data for survivors (13) and deaths (21) [2] and mean ISS data for survivors (8) and deaths (21) [4]. The nature of these studies makes it likely that the investigations included patients with and without trauma activation. Of relevance, authors have stated that ISS ≥16 alone should not be used to identify patients with mortality risk [1].

Decreased GCS with traumatic brain injury has been shown to be associated with in-hospital mortality and unfavorable outcomes post-discharge [5-10]. Four of these studies included patients with GCS 3-15 [5,6,8,9], two studies evaluated patients with GCS 3-12 [5,10], and one study consisted of patients with GCS 3-8 [7]. ICH has also been documented to be associated with unfavorable outcomes post-discharge or hospital mortality [6,9,11-14]. Two of these studies included patients with GCS 3-12 [11,12], three studies consisted of patients with GCS 3-15 [6,9,14], and one investigation included patients with GCS 3-8 [13].

Increased age has also been shown to be associated with in-hospital mortality [15-18]. All of the previous studies described trauma patient results contained within large databases, making it likely that the investigations included patients with and without trauma activation. PEMCs have also been documented to be associated with in-hospital mortality [19-22]. Two of these studies described trauma patient results contained within large databases, also making it likely that the investigations included patients with and without trauma activation [20,21]. One study included trauma patients with ISS ≥16, making it likely that most patients had received trauma activation [22]. One of these studies focused on trauma patients with non-severe injuries (ISS median 6) [19].

Based on ISS and GCS values and their ranges and other inclusion and exclusion criteria in the cited studies, virtually all of these trauma studies either assessed risk conditions in activation patients or activation patients plus non-activation (consultation) patients. These findings indicate that there is minimal evidence for specifically assessing trauma non-activation and consultation patient risk conditions for adverse outcomes. The sole exception was the study that purposely addressed trauma patients with non-severe injuries (median ISS, 6), indicating that the study population was likely to be non-activation patients [19]. This investigation focused on non-ISS injury-based metrics and PEMC associations with mortality. However, this research did not evaluate ISS, GCS, ICH, age, or smoking history as risk conditions for mortality.

One investigation assessing critically ill trauma patients has shown that cigarette smoking was associated with a decreased mortality and a lower proportion of PEMC, except for chronic obstructive pulmonary disease [23]. However, the median age for smokers was only 44.0 years, and it was significantly lower than that for non-smokers. Median (interquartile range) ISS scores in this study suggested that some patients were likely to have undergone trauma activation and that some likely had no activation.

Here, we aimed to assess the statistical associations of three postinjury circumstances (increased ISS, decreased GCS, and presence of ICH) and three preinjury conditions (increased age, PEMC, and smoking history) with death and adverse outcomes in trauma consultation patients. We also sought to determine the accuracy of ISS ≥16 for classifying patients with death and adverse outcomes. Finally, we aimed to assess the relationship between smoking history and PEMC.

## Materials and methods

Ethics statements

The current retrospective study was reviewed by the local institutional review board and determined to be qualified for exemption according to Exempt Category 4 (Bon Secours Mercy Health, IRB number: 2024-Trauma-Dunham). The need for informed consent was waived.

Study design and population

The parent group data source were adult patients admitted to a level I trauma center in Northeast Ohio, from January 21 to July 21 during each year 2018, 2019, and 2020 as described in a previous publication [24]. During this time, the trauma center had a tiered activation system composed of full trauma team activation and partial trauma team activation options.

Inclusion and exclusion criteria

In the current investigation, a consecutive subset of trauma consultation (non-activation) patients aged ≥18 years were included. Patients aged <18 years, those with no trauma center admission, or those with trauma activation were excluded. The total number of patients included was 1,031.

Data collection

Some data were obtained from the trauma registry, and other information was obtained from the electronic medical record. Data from the trauma registry included mechanism of injury, age, GCS, ISS, hospital transfer or scene designation, hospital death, intensive care unit (ICU) stay (days), and hospital stay (days). Information from the electronic medical record included ICH, PEMC score, smoking history, and hospital discharge to hospice. Four inquiries were pursued sequentially in each trauma consultation patient to determine these data results.

The presence or absence of ICH was determined from the brain computed tomography report at admission. Radiology reports were available in three-quarters of the patients. When an initial report was equivocal, the follow-up report was used to confirm or reject the presence of ICH. When there was no brain computed tomography report, the trauma service history, physical examination, and discharge reports were reviewed to confirm the absence of ICH.

Diseases listed in the Charlson Comorbidity Index (CCI) provided the basis for creating a PEMC score for each trauma consultation patient. The CCI uses three score categories: 1-4 points for age ≥50 years, 1-point diseases, and ≥2-point diseases (range, 2-6) (https://www.mdcalc.com/calc/3917/charlson-comorbidity-index-cci) [25]. Age CCI points were not included in the assigned PEMC score. The 1-point diseases were myocardial infarction, congestive heart failure, peripheral vascular (arterial) disease, cerebral vascular accident or transient ischemic attack, dementia, chronic obstructive pulmonary disease, connective tissue disease, peptic ulcer disease, mild liver disease, diabetes mellitus, uncomplicated. The ≥2-point diseases were moderate-severe liver disease, diabetes mellitus with end-organ damage, hemiplegia, moderate-to-severe chronic kidney disease, solid tumor, leukemia, lymphoma, and acquired immunodeficiency syndrome. The PEMC score assignment was based on an electronic medical record review of the emergency department and trauma service history and physical examination records regarding the patient’s medical history. When any one of the ≥2-point diseases existed, a PEMC score of 2 was assigned. Although three of the eight ≥2-point diseases could be scored with a value of 3 or 6, these values were capped at two to decrease the PEMC score range from 0-6+ to 0-2 and simplify the data display. When any two or more of the 1-point diseases existed, a PEMC score of 2 was assigned. When only a single 1-point disease existed, a PEMC score of 1 was assigned. When no CCI disease existed, a PEMC score of 0 was assigned. Thus, the PEMC score range was 0-2.

The categorization of smoking history was determined as follows: Smoking was typed into the electronic medical record search engine panel and submitted. The results were scrolled to align with the date of the trauma center admission. Smoking history was documented as never smoker, past smoker, or current smoker.

To determine hospice discharge, palliative care was typed into the electronic medical record search engine panel and submitted. Any palliative care consultation(s) performed during the trauma center admission were reviewed to determine if the patient was discharged to hospice.

Deaths were the sum of the hospital deaths and patients discharged to hospice. Other investigators of trauma patients with ICH have considered discharge to hospice to be a fatal, terminal event [26]. Adverse outcomes were death, ICU stay ≥2 days, or hospital stay >5 days. Other researchers have identified a need for ICU to represent undertriage in non-activation trauma patients [27]. An electronic medical record audit was performed to determine the principal injury for the patients who died in the hospital or were discharged to hospice.

Statistical analysis

All analyses included all 1,031 trauma consultation patients. Continuous data are presented as the mean±standard deviation, whereas categorical variables are reported as frequency counts and percentages. T-tests were performed to test differences between two groups of continuous data. For dichotomous proportional data conforming to a 2 x 2 contingency table format, the two-tailed Fisher's exact test was employed. The odds ratio (OR) and risk ratio (RR) were computed to quantify proportional differences. For proportional data conforming to a 2 x 3 contingency table format, the chi-square test was employed. Multivariate logistic regression analysis was used to assess independent variable associations relative to dichotomous dependent variables.

Age (mean ± standard deviation) was compared between those surviving and dying (t-test). Proportions of those with a fall mechanism, ISS ≥16, GCS <15, ICH, age ≥ 70 years, PEMC, and smoking history were compared between those surviving and dying (two-tailed Fisher's exact test). Proportions of those with PEMC scores of 0, 1, and 2 were compared between those surviving and dying (chi-square test). Stepwise multivariate regression analysis delineated whether these conditions (fall mechanism, ISS ≥16, GCS <15, ICH, age, age ≥ 70 years, PEMC, smoking history, and PEMC score 0-2) had an independent association with death. The proportions of those with survival or death relative to ISS ≥16 or ISS <16 were computed (two-tailed Fisher's exact test). An area under the receiver operating characteristic curve was computed to describe this relationship.

Proportions of those with a fall mechanism, ISS ≥16, GCS <15, ICH, age ≥ 70 years, PEMC, and smoking history were compared between those without and with adverse outcomes (two-tailed Fisher's exact test). Proportions of those with PEMC scores of 0, 1, and 2 were compared between those without and with adverse outcomes (chi-square test). Stepwise multivariate regression analysis delineated whether these conditions (fall mechanism, ISS ≥16, GCS <15, ICH, age ≥ 70 years, PEMC, smoking history, and PEMC score 0-2) had an independent association with adverse outcomes. The proportions of those without or with adverse outcomes relative to ISS ≥16 or ISS <16 were computed (two-tailed Fisher's exact test). An area under the receiver operating characteristic curve was computed to describe this relationship.

Age (mean ± standard deviation) was compared between those without and with a PEMC (t-test). Proportions of those with a smoking history were compared between those without and with a PEMC (two-tailed Fisher's exact test). Multivariate regression analysis was performed to determine if age and smoking history had independent associations with PEMC.

Data were entered into Excel 2010 (Microsoft Corp., Redmond, WA, USA) and imported into SAS System for Windows (release 9.2, SAS Institute Inc., Cary, NC, USA). For receiver operating characteristic curve analyses, data were exported from SAS into MedCalc® Statistical Software (version 22.016, MedCalc Software Ltd., Ostend, Belgium). The significance level for the p-value was set at <0.05.

## Results

The parent group consisted of 2,076 consecutive trauma center admissions studied over an 18-month period (January 21 to July 21 during each year 2018, 2019, and 2020) [24]. The current study focused on 1,031 trauma consultation, non-activation patients. The patient characteristics are presented in Table 1. Most patients had a fall mechanism, one-half were aged ≥70 years, one-half had a PEMC, and proportions of ISS ≥16 and ICH were substantial.

**Table 1:** Characteristics of the trauma consultation patients Data are presented as mean ± standard deviation or n (percentage).

Category	Total (n=1031)
Injury Mechanism	
Fall	774 (75.1%)
Motorized crash	154 (14.9%)
Penetrating injury	7 (0.7%)
Other	96 (9.3%)
Injury Severity	
Injury Severity Score ≥16	218 (21.1%)
Glasgow Coma Scale (GCS) score <15	143 (13.9%)
Intracranial hemorrhage	342 (33.2%)
Age	
Mean age	65.4 ± 21
Age ≥70 years	508 (49.3%)
Preexisting Medical Conditions (PEMC)	
PEMC score 0	498 (48.3%)
PEMC score 1	241 (23.4%)
PEMC score 2	292 (28.3%)
PEMC score 1 or 2	533 (51.7%)
Smoking History	
Never smoker	490 (47.5%)
Current smoker	272 (26.4%)
Former smoker	269 (26.1%)
Hospital Transfer	
Hospital transfer	630 (61.1%)
Hospital death plus hospice discharge	28 (2.7%)
Intensive care unit	156 (15.1%)
Hospital stay (days)	3.9 ± 3.4
Hospital stay >5 days	210 (20.4%)

Of the 28 cases categorized as a death, 25 patients died before discharge from the trauma center and three were transferred from the trauma center to hospice. Univariate associations with death are shown in Table 2. Death was associated with ISS ≥16, GCS <15, age ≥70 years, and PEMC score 1 or 2, where the measures of association (OR and RR) were substantial. Death was also associated with ICH; however, the measures of association were not as great. Of the 28 deaths, ICH was present in 16 (57.1%). Those with a smoking history had a mean age of 61.9±20 years and a median age of 64.0. The proportion of ISS ≥16 was lower in patients without ICH (8.7% (60/689)) than in those with ICH (46.2% (158/342); p<0.0001; OR=9.0). Multivariate logistic regression analysis demonstrated that death had independent associations with increasing age (p=0.0019), decreasing GCS (p<0.0001), ISS ≥16 (p=0.0074), and PEMC (p=0.0137). ICH did not have an independent association with death when considered among the other four variables.

**Table 2:** Univariate associations with death ╠ 2 x 3 chi-square analysis; all other proportional analyses were two-tailed Fisher's exact test. χ^2^: chi-square value; OR: odds ratio; RR: risk ratio; ISS: Injury Severity Score; GCS: Glasgow Coma Scale score; ICH: intracranial hemorrhage; SD: standard deviation; PEMC: preexisting medical condition

Variable	Death - No	Death - Yes	p-value	χ^2^	OR	RR	t-value
Total	1,003 (97.3%)	28 (2.7%)	–	–	–	–	–
Fall mechanism	747 (74.5%)	27 (96.4%)	0.0081	7.0	9.3	1.3	–
ISS ≥16	203 (20.2%)	15 (53.6%)	<0.0001	18.2	4.6	2.7	–
GCS <15	131 (13.1%)	12 (42.9%)	<0.0001	20.2	5.0	3.3	–
ICH	326 (32.5%)	16 (57.1%)	0.0063	7.5	2.8	1.8	–
Age (mean ± SD)	64.9 ± 21	83.1 ± 13	<0.0001	–	–	–	-7.24
Age ≥70 years	483 (48.2%)	25 (89.3%)	<0.0001	18.4	8.8	1.9	–
PEMC score 0	494 (49.3%)	4 (14.3%)	–	–	–	–	–
PEMC score 1	234 (23.3%)	7 (25.0%)	–	–	–	–	–
PEMC score 2	275 (27.4%)	17 (60.0%)	0.0002	17.6^╠^	–	–	–
PEMC score 1 or 2	509 (50.8%)	24 (85.7%)	0.0002	13.3	5.8	1.7	–
Smoking history	527 (52.5%)	14 (50.0%)	0.7905	0.1	–	–	–

A 2 x 2 contingency table was constructed to describe the relationship between death and ISS ≥16 and ISS <16 (Table 3). The cross-tabulation matrix revealed the following counts: true positive, 15; false negative, 13; false positive, 203; and true negative, 800. Derivative values were as follows: sensitivity, 53.6%; specificity, 79.8%; accuracy, 79.0%; positive-predictive value, 6.9%; negative-predictive value, 98.4%; and area under the receiver operating characteristic curve, 0.7 (p<0.001). From a perspective standpoint relative to death, 93.1% (203/218; 95% confidence interval: 88.7-96.0%) of patients with an ISS ≥16 survived and 46.4% (13/28) of those who died had an ISS <16.

**Table 3:** Relationships of death and survival with Injury Severity Score ≥16

Category	Death	Survival
Injury Severity Score ≥16	15	203
Injury Severity Score <16	13	800

Of the 28 deaths, the numbers of principal injuries were as follows: ICH in 15 cases; both ICH and spine injury in one case; spine injury alone in nine cases; rib fractures in two cases; and sternal fracture in one case. Of the 16 deaths with ICH, the pathologies were subdural hematoma in 12, intracerebral hematoma in one (nine of these 13 had a mass effect), and subarachnoid hemorrhage in three. Of these 16 deaths, the following characteristics were found: age, 82.4±15 years; ISS, 21.3±7.3; PEMC score 1 or 2, 12 (75.0%); and age ≥70 years or PEMC score 1 or 2, 15 (93.8%).

Univariate associations with adverse outcomes are shown in Table 4. Adverse outcomes were associated with ISS ≥16, GCS <15, ICH, and PEMC. Multivariate logistic regression analysis demonstrated that adverse outcomes had independent associations with a decreasing GCS (p<0.0001), ISS ≥16 (p<0.0001), and PEMC (p=0.0005). ICH did not have an independent association with adverse outcomes when considered among the other three variables.

**Table 4:** Univariate associations with adverse outcomes ╠ 2 x 3 chi-square analysis; all other proportional analyses were two-tailed Fisher's exact test. AO: adverse outcomes; χ^2^: chi-square value; OR: odds ratio; RR: risk ratio; ISS: Injury Severity Score; GCS: Glasgow Coma Scale score; ICH: intracranial hemorrhage; PEMC: preexisting medical condition

Variable	AO - No	AO - Yes	p-value	χ^2^	OR	RR
Total	773 (75.0%)	258 (25.0%)	-	-	-	-
Fall mechanism	551 (71.3%)	223 (86.4%)	<0.0001	23.7	2.5	1.2
ISS ≥16	117 (15.1%)	101 (39.2%)	<0.0001	66.9	3.5	2.6
GCS <15	82 (10.6%)	61 (23.6%)	<0.0001	27.5	2.5	2.2
ICH	232 (30.0%)	110 (42.6%)	0.0004	13.9	1.7	1.4
Age ≥70 years	368 (47.6%)	140 (54.3%)	0.0640	3.4	–	–
PEMC score 0	400 (51.6%)	98 (38.3%)	–	–	–	–
PEMC score 1	180 (23.2%)	61 (23.8%)	–	–	–	–
PEMC score 2	193 (25.0%)	99 (38.4%)	0.0001	19.9^╠^	–	–
PEMC score 1 or 2	373 (48.3%)	160 (62.0%)	0.0002	14.7	1.7	1.3
Smoking history	404 (52.3%)	137 (53.1%)	0.8157	0.1	–	–

A 2 x 2 contingency table describing the relationship between adverse outcomes and ISS ≥16 and ISS <16 is shown in Table 5. The cross-tabulation matrix revealed the following counts: true positive, 101; false negative, 157; false positive, 117; and true negative, 656. Derivative values were as follows: sensitivity, 39.1%; specificity, 84.9%; accuracy, 73.4%; positive-predictive value, 46.3%; negative-predictive value, 80.7%; and area under the receiver operating characteristic curve, 0.6 (p<0.001). From a perspective standpoint relative to adverse outcomes, 53.7% (117/218) of patients with an ISS ≥16 did not have an adverse outcome, and 60.9% (157/258) of patients with an ISS <16 had adverse outcomes.

**Table 5:** Relationships of the presence and absence of adverse outcomes with Injury Severity Score ≥16

Category	Adverse Outcome	No Adverse Outcome
Injury Severity Score ≥16	101	117
Injury Severity Score <16	157	656

Any PEMCs were present in 533 (51.7%) and absent in 498 (48.3%) trauma consultation patients. Age was greater with PEMC (75.3±14 years) than without PEMC (54.9±2 years; p<0.0001). The proportion of past or current smoking history was greater with PEMC (305 (57.2%)) than without PEMC (236 (47.4%); p=0.0016). Multivariate logistic regression analysis found that any PEMC had independent associations with a past or current smoking history (p<0.0001) and age (<0.0001). The r-square for the multivariate model was 0.3.

## Discussion

Association of ISS ≥16 with death

Although ISS ≥16 in trauma consultations had a significant association with death in the current study, its classification precision was marginal. The borderline classification accuracy of ISS ≥16 with death is objectively exemplified by the low value for the area under the receiver operating characteristic curve. In this regard, it is important to note that over 90% of patients with ISS ≥16 survived and 50% of those who died had an ISS <16. Other investigators have also shown that the proportion of trauma patient consultation deaths among patients with ISS ≥16 has been less than 10% [28-30]. Of relevance, one investigation has shown that an increased ISS had an association with mortality in undertriaged trauma patients; however, the OR was small [31]. Another investigation that examined age ≥65 years with ISS ≥16 found that non-activation patients had a better discharge outcome than activation patients [32].

Other researchers have recognized the limitations of ISS ≥16 for assessing undertriage outcomes by qualifying ISS ≥16 patients with a need for trauma interventions [29,30,33-35]. These findings bring into question the validity of classifying non-activation patients by ISS ≥16 as a reliable method for portending adverse outcomes. Several investigations have shown that the severity of anatomic injury, using the ISS, is associated with in-hospital mortality [1-4]. However, these studies, based on their inclusion criteria, likely consisted of trauma activation patients or activation patients plus non-activation patients. We believe that trauma consultation patient risk and outcome validity would be enhanced if analyses were conducted solely on that cohort.

Association of GCS <15 with death

The present study found that GCS <15 was associated with death. Decreased GCS with traumatic brain injury is associated with in-hospital mortality and unfavorable outcomes post-discharge [5-10]. However, these studies, based on their investigation criteria, likely consisted of trauma activation patients or activation plus non-activation patients. One investigation of undertriaged trauma patients also found that a decreased GCS was associated with mortality [31].

Association of ICH with death

Here, ICH was found in one-third of trauma consultation patients, and it had an association with mortality. Importantly, ICH was present in over 50% of the non-activation patients who died. ICH has also been found by others to be common in non-activation elderly trauma center patients [36]. Furthermore, ICH has been documented to be associated with hospital mortality and unfavorable outcomes post-discharge [6,9,11-14]. However, these studies, based on their inclusion criteria, likely consisted of trauma activation patients or activation patients plus non-activation patients. One study of undertriaged trauma patients with ISS ≥16 found that most had ICH [37]. In a study of inter-hospital transfers with ICH to a trauma center, one-fifth of the patients were managed as a consultation [26]. The aforementioned evidence indicates that ICH is a common phenomenon in trauma consultation patients.

Association of age ≥70 years with death

In the present study, age ≥70 years had a large, significant association with death. Increased age has also been shown by multiple other investigators to be associated with in-hospital mortality [15-18]. Because all of these studies described results of trauma patients from large databases, it is likely that the investigations included patients with and without trauma activation. Increased age has been identified by other investigators to be associated with undertriage [38]. In a study of patients with ISS ≥16 and age ≥65 years, the cohort without activation was older than that with activation [39]. To our knowledge, the current study is the only investigation of trauma consultation patients to demonstrate that age ≥70 years has a significant association with death.

Association of PEMC with death

The current study showed that PEMC had a substantial association with death. Several others have demonstrated that PEMC is associated with in-hospital mortality [19-22]. However, these studies, based on their study criteria, likely consisted of trauma activation patients or activation plus non-activation patients. Of relevance, another publication on trauma center inter-hospital transfers with ICH showed that many patients had PEMC [26]. Two studies have shown that PEMC is common in elderly trauma center patients [40,41]. Similar findings have been demonstrated in non-activation elderly trauma center patients [36].

In a study of patients with ISS ≥16 and age ≥65 years, the cohort without activation had a higher PEMC proportion than those with activation [39]. Of importance, a study of ISS ≥16 patients without activation showed that those with unfavorable outcomes had a higher PEMC proportion than those with favorable outcomes [32]. These studies and the current study support the notion that PEMC are common in non-activation, consultation patients and are associated with death.

Association of smoking history with death

Patients in the present study with a smoking history had neither a direct nor inverse association with death. This is in contradistinction to findings of an investigation of critically ill trauma patients where those with a smoking history had a lower mortality than patients without a smoking history [23]. However, the median age for smokers was only 44.0 years and was significantly lower than that for non-smokers. Median (interquartile range) ISS scores in this study suggested that some patients were likely to have undergone trauma activation whereas others were likely to have had no activation. These two findings contrast with the current study findings in that smokers had a substantially greater median age of 64.0 years and that all of the patients were without trauma activation.

Independent associations with death

Death had independent associations with increasing age, decreasing GCS, ISS ≥16, and PEMC. The observation that three conditions, besides ISS ≥16, point to the relative imprecision of ISS ≥16 alone to properly classify dying patients. That is, other conditions besides the magnitude of anatomic injury influence the propensity to die. In a relevant publication, the authors specifically stated that ISS ≥16 alone should not be used to identify patients with mortality risk [1]. Another investigation of likely non-activation trauma patients demonstrated the importance of combining PEMC status with an injury-based metric to enhance the association with mortality [19]. Of the dying patients in the current study, virtually all were aged ≥70 years and had PEMC; more than one-third had GCS <15; and most had ICH. Because the dependent variable of interest, death, in the current study occurred in <5% of 1,000 patients, statistically significant associations with risk conditions should be considered to be robust and compelling evidence.

It is important to consider the reason why ICH was not found to be an independent risk condition for death. Univariate analysis demonstrated that the proportion of ISS ≥16 was much greater in patients with ICH than in those without ICH. That is, ICH would be considered statistically irrelevant if the ISS ≥16 status is included in the analysis. However, it is clinically important to realize that over 50% of the patients dying had ICH. Further mitigating the lack of independence of ICH in the multivariate analysis is that over 90% of the patients who died with ICH were aged ≥70 years or had PEMC.

Associations of adverse outcomes

Adverse outcomes were defined as death, ICU stay of two days or more, or hospital stay exceeding five days; this information is readily available from any trauma registry. Associations of adverse outcomes were analogous to those for death with some exceptions. Adverse outcomes were associated with ISS ≥16, ICH, GCS <15, and PEMC, but not age ≥70 years. Multivariate logistic regression analysis demonstrated that adverse outcomes had independent associations with ISS ≥16, decreasing GCS, and PEMC. This analysis also indicated that conditions besides ISS ≥16 influence adverse outcomes. ICH did not have an independent association with adverse outcomes for the same reasons previously discussed for death. Trauma center personnel with oversight authority should consider qualifying ISS ≥16 non-activation patients with adverse outcomes as those who might represent potential undertriage.

Association of smoking history and PEMC

Univariate analysis in the present study demonstrated that consultation patients with any PEMC were older and had a higher proportion of current or past smoking history at the time of trauma center admission. Increased age and positive smoking history were found to have independent associations with any PEMC. The r-square for this independent association was compelling. This is in contradistinction to results of an investigation of critically ill trauma patients where those with a smoking history had fewer proportions of comorbidities than patients without a smoking history [23]. However, the median age for smokers was only 44.0 years and was significantly lower than that for non-smokers. The ISS in that study suggested that some patients were likely to have undergone trauma activation whereas others were likely to have had no activation. These two findings contrast with the results of the current study where smokers had a substantially greater median age and that all of the patients were without trauma activation. Although not trauma studies, other investigators have shown that smokers have higher CCIs than non-smokers [42,43].

Limitations of the study

The principal limitation of the current study is its retrospective design. Its findings need to be compared with data that are prospectively collected and support a specific set of hypotheses. The PEMC score in this study does not precisely reflect the values given in most publications on the CCI. Another limitation is that the ISS was the only anatomic injury descriptor used, although other anatomic injury assessments exist. Another consideration is the data used in the current analysis included the COVID-19 pandemic period and did not include the entire calendar years.

## Conclusions

A conventional ISS cut-point is marginally accurate for classifying trauma consultation patients at risk for death and adverse outcomes. Besides ISS, other postinjury conditions and preinjury traits should be considered when assessing trauma consultation patient risk for death and adverse outcomes. Trauma consultation patients with increased ISS and trauma registry-derived adverse outcomes should be considered as a cohort that might have been undertriaged. To the best of our knowledge, this is the only investigation that has included scene and hospital transfer trauma consultation patients and produced an analysis of multiple preinjury and postinjury risk conditions.
